# Feed- and feed additives-related aspects of gut health and development in weanling pigs

**DOI:** 10.1186/2049-1891-4-1

**Published:** 2013-01-07

**Authors:** John R Pluske

**Affiliations:** 1 Division of Research and Development, Murdoch University, South Street Murdoch, WA, 6150, Australia

**Keywords:** Antibiotics, Antimicrobials, Nutrition, Pigs, Post-weaning diarrhoea, Weaning

## Abstract

The development of new/different management and feeding strategies to stimulate gut development and health in newly-weaned pigs, in order to improve growth performance while minimizing the use of antimicrobial compounds such as antibiotic growth promotants (AGP) and heavy mineral compounds, is essential for the long-term sustainability of the pig industry. Factors including the sub-optimal intake of nutrients and energy, inappropriate microbiota biomass and (or) balance, immature and compromised immune function, and psychosomatic factors caused by weaning can compromise both the efficiency of digestion and absorption and intestinal barrier function through mucosal damage and alteration of tight junction integrity. As a consequence, pigs at weaning are highly susceptible to pathogenic enteric conditions such as post-weaning diarrhea that may be caused by serotypes of enterotoxigenic *Escherichia coli*. Many dietary components, e.g., protein, fiber, feed additives and minerals, are known to influence microbial growth in the gastrointestinal tract that in turn can impact upon pig growth and health, although the relationships between these are sometimes not necessarily apparent or obvious. In a world climate of increased scrutiny over the use of antibiotics *per se* in pig production, certain feed additives are seen as alternatives/replacements to antibiotics, and have evolved in some cases to have important roles in everyday commercial pig nutrition. Nevertheless and in general, there remains inconsistency and variability in the efficacy of some feed additives and in cases of severe disease outbreaks, for example, therapeutic antibiotics and/or heavy minerals such as zinc oxide (ZnO) are generally relied upon. If feed ingredients and (or) feed additives are to be used with greater regularity and reliability, then it is necessary to better understand the mechanisms whereby antibiotics and minerals such as ZnO influence animal physiology, in conjunction with the use of appropriate challenge models and *in vitro* and *in vivo* techniques.

## Introduction

Weaning under modern-day commercial conditions inflicts stress (environmental, nutritional, psychological/social) on pigs and is associated with marked changes in gastrointestinal tract (GIT) physiology, microbiology and immunology [1,2]. Consequently the period following weaning is generally characterized by sub-optimal growth (e.g., low feed intake, body weight loss); [1,3], deteriorated feed efficiency, and a high incidence of intestinal disturbances with diarrhea (of bacterial and (or) dietary origin) often occurring that, in turn, can cause morbidity and/or mortality [4-6]. Indeed, under practical commercial conditions and both before and after weaning, young pigs probably achieve less than 50% of their growth performance potential [1].

To assist in overcoming the post-weaning growth check, antibiotics and (or) some mineral compounds such as ZnO have traditionally been included in diets for weanling pigs [7]. However and owing to the possible contribution of in-feed antibiotics to the development of antibiotic-resistant strains of bacteria [8-10], the European Union implemented a full ban on in-feed antibiotics usage in livestock diets in January 2006. There is also pressure in other pig-producing regions of the world to minimize or completely eliminate the inclusion of in-feed antibiotic in livestock diets, for a number of different reasons [11,12]. There are also concerns about environmental accumulation of minerals.

Consequences for the removal of AGPs include a reduction of livability, a decrease in body weight, a decrease in feed conversion efficiency, less uniformity (i.e., more variation within a pen of pigs) and increased use of therapeutic antibiotics. To improve the productivity, health and welfare of pigs in the post-weaning period, it is necessary to find combinations of feed ingredients, either alone or in combination with feed additives acceptable for use, that are effective in ameliorating the post-weaning growth check and reducing the incidence and severity of digestive problems frequently encountered. In this regard, a better understanding of the mechanisms whereby antibiotics influence animal physiology, as well as appropriate use of disease models and *in vitro* techniques, will lead to the development of alternatives to current antimicrobial compounds. Given the considerable advances already made in the understanding of intestinal nutrient utilization and metabolism, de Lange *et al*. [13] commented that a complimentary goal in nutrition might be to formulate young pig diets with the specific task of optimizing the growth, function and health of the GIT, for example addressing the protein/amino acid content of diets, minimal buffering capacity, minimal content of anti-nutritional factors, and supply of beneficial compounds, for example, growth factors and immunoglobulins. The optimum dietary level and type of fiber will also vary according to the nature of enteric disease challenges, ingredient supply and cost, and the production objectives [13].

A large and continuously growing body of research exists evaluating the impact of a wide range of feed ingredients and feed additives on various aspects of GIT health and development in pigs. This cannot be reiterated to any great detail in this paper, but rather, the aim of this paper is to briefly summarize the main issues interrelating the GIT after weaning to health and development, a brief description of feed additives, and then a specific exploration of post-weaning diarrhea in relation to feedstuffs and feed additives.

## The gastrointestinal tract (GIT) after weaning and its relationship to health and development

**Figure 1:**
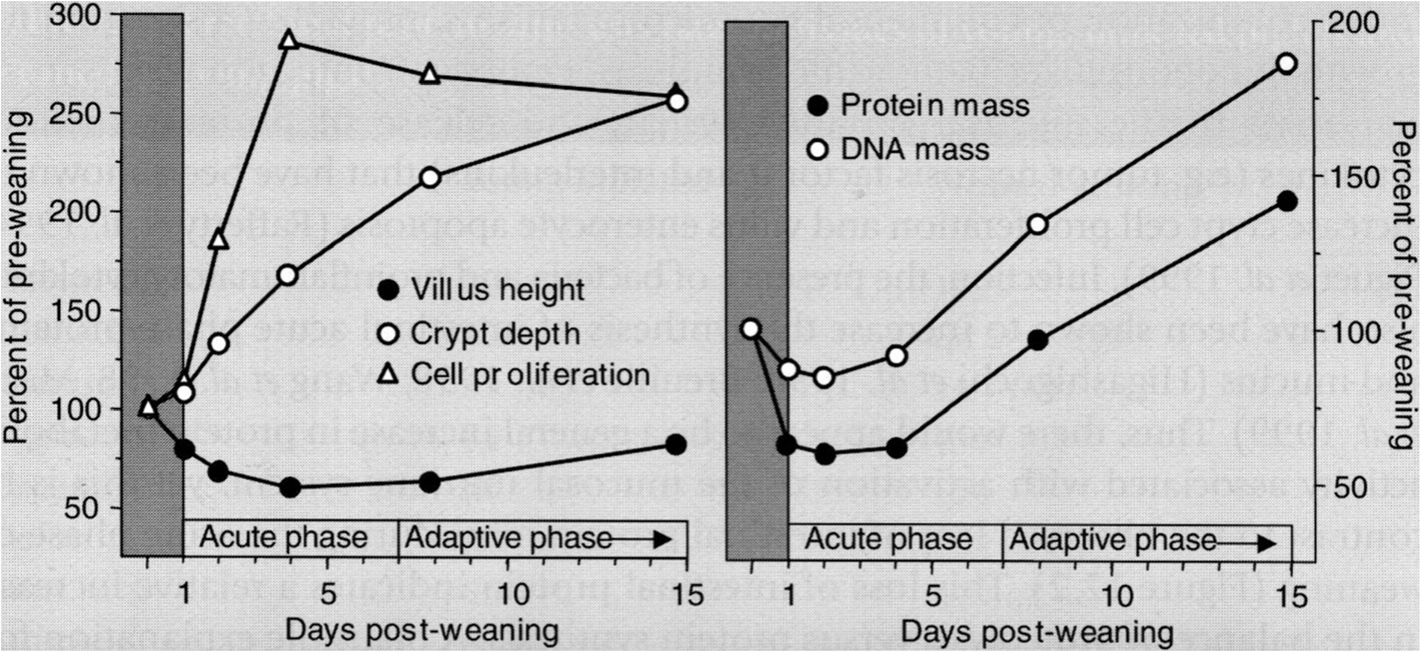
**The acute and adaptive phases in development of earlyweaned pigs **[14]**.**

The GIT of a pig is a very complex environment, with the GIT of young pigs around the time of weaning undergoing rapid changes in size, protein turnover rates, microbiota mass and composition, and quick and marked alterations in digestive, absorptive, barrier and immune functions [1,14-17]. Burrin and Stoll [14] divided these changes into the *acute phase*, observed within the first 5–7 days after weaning, and the *adaptive phase*, which occurs after this (Figure 1). These workers distinguish between the acute and adaptive phases based primarily on the changes in feed intake and the subsequent impacts that enteral (luminal) nutrition has on the GIT, because it takes 7–14 days for weaned pigs to learn how to eat and resume a level of dry matter intake (at least) that is comparable to that during the pre-weaning period [1]. If the GIT is deficient in macronutrients, micronutrients and energy, then its health, development and any subsequent recovery in the adaptive phase will be impaired.

As stated by de Lange *et al*. [13], it is naïve to suggest that given these complexities and rapid changes after weaning, a limited number of feed additives can be effective in stimulating gut development and health in different groups of pigs that are managed under wide ranging conditions of housing, management, feeding and health status. This emphasizes the need to explore underlying mechanisms when evaluating the functional properties of feed ingredients and feed additives, so that we may better understand under what conditions we can achieve the optimal response to dietary interventions. Key aspects of gut functionality that should be considered include digestive capacity (activity of pancreatic and brush-border enzymes), absorptive capacity, chemical and physical barriers, microbiota load and diversity, and immune function [13], and within this context, it is surprising how limited our understanding is of how antibiotics interact with gut tissue, either directly or indirectly via microbiota and fermentation products [18]. In contrast, ZnO is purported to have a wide variety of modes of action [19] making it difficult to interpret what the precise mechanism is (or precise mechanisms are) for its efficacy; this makes the search/development of products to replace ZnO, if indeed that needs to occur, more problematic. Regardless, such understanding is critical for finding effective, sustainable and consumer-acceptable replacements for these products.

With the objective being to improve productivity of pigs managed under commercial conditions, it is generally a challenge for researchers to obtain reliable experimental data from commercial pig units that will allow for a detailed exploration of the underlying mechanisms or to evaluate a wide range of feed additives. Alternatively, it can be a challenge to properly represent commercial conditions in research units [13]. In this sense, the use of *in vitro* techniques and infectious disease models may be considered as well. For example, apparatus such as Ussing chambers may be used to evaluate various aspects of mucosal functionality - such as permeability, absorptive capacity and secretary function - allowing a rapid screening of feed additives and feed ingredients [20].

Alternatively, the antimicrobial properties of a wide range of feed additives may be explored in relatively simple *in vitro* systems although care should be taken with the interpretation of such findings. For example, the *invitro* determined anti-microbial properties of various essential oils are diminished substantially when these are determined with the presence of feed in the *in vitro* system [21], most probably because essential oils are adsorbed quickly to feed particles. Observations made *in vitro* therefore need to be substantiated with key measurements made *in vivo*.

## Feed additives and antibiotics

The Official Journal of the European Union [22] defines feed additives as substances, micro-organisms or preparations, other than feed material and premixtures, which are intentionally added to feed or water in order to perform, in particular, one or more of the following functions:

•favorably affect the characteristics of feed,

•favorably affect the characteristics of animal products,

•favorably affect the colour of ornamental fish and birds,

•satisfy the nutritional needs of animals,

•favorably affect the environmental consequences of animal production,

•favorably affect animal production, performance or welfare, particularly by affecting the gastro-intestinal flora or digestibility of feedingstuffs, or

•have a coccidiostatic or histomonostatic effect.

In this regard, the feed additive shall *not*:

•have an adverse effect on animal health, human health or the environment,

•be presented in a manner which may mislead the user,

•harm the consumer by impairing the distinctive features of animal products or mislead the consumer with regard to the distinctive features of animal products [22].

Antibiotics, other than coccidiostats or histomonostats, shall not be authorized as feed additives [22].

Nevertheless, antibiotics are vital medicines used for the treatment of bacterial infections in both humans and animals, however and as mentioned previously, it is the emergence of antibiotic resistance as a serious problem in human medicine that has prompted concerns about the public health implications of antibiotic use in animal agriculture. Antibiotics have been used for over 40 years in farm animals for 3 main purposes:

1. Therapy, to treat an identified illness

2. Prophylaxis, to prevent illness in advance

3. Performance enhancement, to increase feed conversion, growth rate or yield [23].

**Figure 2:**
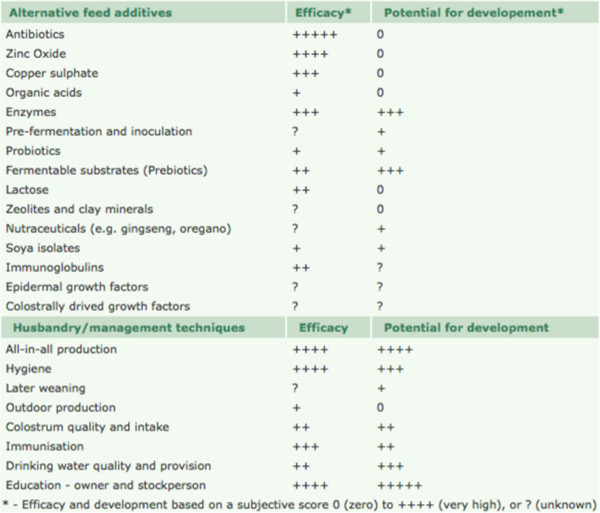
**The efficacy and potential for developing alternative additives and strategies to replace the role of antibiotic feed additives in pig production **[23]**.**

Currently it is unlikely that there is any single substance that could replace the function of feed antibiotics [13,19]. Moreover, any single substance that is intended to wholly replace the role of antibiotics in farm animals will be subject to the intense scrutiny that antibiotics have been subjected to over the past 40 years. Since the growth benefit found from feeding antibiotics is achieved through many different effects on the GIT, the strategy for replacing them will depend on a combination of nutritional, management, housing, health and (or) husbandry factors. There is also considerable inconsistency in the experimental and (or) commercial outcomes of the many alternatives evaluated, which makes it difficult to judge the efficacy or otherwise of a particular additive. Nevertheless, an attempt has been made to evaluate the efficacy of the many individual feed additives promoted as alternatives to antibiotics (Figure 2), with a score to give an indication of their potential value [23].

As can be seen, these authors have included husbandry and management techniques in their evaluation, and interestingly rated some of the factors (e.g., education for the owner and stockpersons) as having the greatest potential for development in the absence of antibiotics. It is imperative, therefore, that a holistic standpoint be considered in this topic.

## Post-weaning diarrhea, feed, and feed additives: a specific example

Post-weaning diarrhea (PWD) is a condition in weaned pigs characterized by frequent discharge of watery feces during the first 2 weeks after weaning and continues to represent one of the major economic problems for the pig industry worldwide, causing widespread morbidity and/or mortality in the most serious of affected cases. Post-weaning diarrhea is a multi-factorial disease, and its precise pathogenesis is still unclear, however it is typically associated with fecal shedding of large numbers of **β**-hemolytic enterotoxigenic *E*. *coli* serotypes that particularly proliferate in the small intestine after weaning, and for this reason, PWD is sometimes also called post-weaning colibacillosis [23]. Other pathogenic types of *E*. *coli* that are not enterotoxigenic occasionally may be involved in PWD, and there are many different types of bacterial pilus (fimbrial) adhesins that may be involved in attachment to the intestinal mucosa [24].

Antibiotic growth promoters (AGP) have long been used not only for elimination or reduction in pathogenic bacteria but also for promotion of growth, with an obvious and key target for AGP being enterotoxigenic *E*. *coli* in the post-weaning period. However and as described previously, restrictions/concerns on their use have seen many of the alternatives, such as feed additives and dietary interventions, been proposed for use in alleviating the problems encountered with PWD. In this regard, PWD represents an interesting case-in-point because it is a condition, in the absence of AGP, which can be approached using feed (e.g., diet composition, protein/amino acid levels) and/or feed additives (e.g., ZnO, where permitted, organic acids) as possible solutions.

Recently, Heo *et al*. [6] provided a very good overview of the various options available for producers concerning PWD, with Halas *et al*. [5], de Lange *et al*. [13] and Kim *et al*. [25] also contributing to understanding and knowledge in this area. It is recognized, for example, that both the source and level of dietary protein are known to influence enteric health in piglets, and a considerable body of research dating back to the 1950s and 1960s has implicated an association between protein level in the diet and diarrhea after weaning. A key component of this association to PWD is the amount and type of fiber present in the diet, and numerous studies have investigated relationships between the amount and type of both dietary carbohydrate and protein on GIT characteristics and PWD [26,27]. Kim *et al*. [28] showed that clinical expression of PWD could be dependent on the balance of fermentable carbohydrates and proteins available in the GIT rather than absolute amount of protein or non-starch polysaccharides (NSP) in the digesta. In this study, the authors fed diets containing 190–200 g protein/kg diet that were based on extruded rice or raw wheat without and with 20 g/kg oat hulls. The protein sources were all animal proteins to limit other sources of NSP in the diet. The basal diets without oat hulls contained 3 g and 11 g soluble NSP and 9 g and 66 g insoluble NSP/kg diet, respectively for extruded rice and raw wheat-based diets. Interestingly, expression of PWD was higher only in pigs fed an extruded rice-based diet without oat hull supplementation while the pigs fed a wheat-based diet without oat hulls did not develop PWD. This interaction may possibly indicate that the ratio between fermentable protein and carbohydrates in the GIT could affect the development of PWD.

Studies have also evaluated the influence of dietary protein feeding on the composition of the microbiota, however and as highlighted by Heo *et al*. [6], further research is required investigating the impacts of the optimum fermentable protein/fermentable carbohydrate ratio on intestinal bacterial characteristics to understand more clearly their impact on the microbial ecology of the GIT in weaned pigs. In this way, practical dietary solutions can be obtained. Nevertheless, a consistent way to reduce the incidence and severity of PWD appears to be feeding of a lower-protein diet, and indeed, offering a lower-protein diet with essential amino acid supplementation (lysine, methionine, threonine, tryptophan, valine and isoleucine) or appropriate ingredients, and for as little as 5 days after weaning [29], improves indicators of GIT health in piglets without imposing a production loss. Furthermore, recent work by Bhandari *et al*. [30] suggested that feeding a low-protein diet could also act synergistically with other nutritional interventions to promote growth performance in piglets. These authors reported that a low-protein diet acted synergistically with probiotics (non-pathogenic *E*. *coli* probiotics) to improve growth performance in piglets compared with in-feed antibiotics (Table 1). Supplementation of pig starter diets with probiotics is recognized as one of the non-pharmaceutical alternatives/replacements to antibiotics in weaned pig diets.

**Table 1:** **Performance and fecal consistency (FC) score of early-weaned pigs fed different diets**[29]

**Item**	**Diets**^**a**^									
	**HP**			**LP**				***P*-value**^**c**^		
	**NA**	**AB**	**PRO**	**NA**	**AB**	**PRO**	**SEM**^**b**^	**A**	**B**	A*B
Initial BW, kg	6.74	6.67	6.74	6.67	6.83	6.73	0.348	0.943	0.985	0.992
Final BW, kg	8.24	8.29	8.42	8.60	8.60	9.30	0.379	0.104	0.438	0.715
ADG, g										
Pre-infection, d 0 to 6	73	94	79	121	101	179	8.41	<0.001	<0.001	<0.001
Post-infection, d 7 to 12	176	176	200	201	219	249	16.33	0.007	0.086	0.757
ADFI, g										
Pre-infection, d 0 to 6	123	140	122	187	128	238	41.56	0.111	0.480	0.363
Post-infection, d 7 to 12	288	261	287	299	281	326	34.11	0.384	0.624	0.917
G:F, g/g										
Pre-infection, d 0 to 6	0.43	0.56	0.51	0.47	0.59	0.73	0.048	0.088	0.179	0.460
Post-infection, d 7 to 12	0.56	0.70	0.65	0.57	0.68	0.73	0.060	0.508	0.101	0.643
FC score^d^										
6 h	1.38	1.15	1.04	1.00	0.80	0.91	0.071	<0.001	0.008	0.180
24 h	2.04	1.45	1.38	1.67	1.10	1.29	0.113	0.009	<0.001	0.382
48 h	2.35	1.60	1.63	1.83	1.35	1.29	0.100	<0.001	<0.001	0.412
72 h	2.38	1.55	1.42	1.75	1.05	1.17	0.086	<0.001	<0.001	0.103
96 h	1.88	1.15	1.04	1.45	0.90	0.88	0.117	0.009	<0.001	0.564
120 h	1.29	0.75	0.70	1.00	0.70	0.63	0.122	0.162	<0.001	0.582

^a^ HP = high protein; LP = low protein; NA = no additive; AB = in-feed antibiotics; PRO = probiotics (UM 2 and Um 7).

^b^ Pooled SEM; *n* = 6/diet.

^c^ A = factor A - two levels of protein (HP and LB); B = factor B - three additive (NA, AB, and PRO).

^d^ Fecal consistency score: 0, normal; 1, soft feces; 2, mild diarrhea; and 3, severe diarrhea.

In this particular study, the low protein diet reduced *E*. *coli* K88 counts on the mucosa surface with a concomitant reduction in *E*. *coli* K88 counts in the digesta. The improved performance observed with probiotic supplementation was probably mediated via a reduction of *E*. *coli* K88 colonization of the mucosal surface and subsequently the reduction of PWD, as indicated by the fecal score (FC) results (Table 1). These authors explained that the synergistic effect between the low protein diet and the probiotic used in the current study on post-weaning performance of piglets was probably a result of different mechanisms by which they control *E*. *coli* K88 populations, with the low protein diet reducing the amount of substrate available for *E*. *coli* K88 proliferation [31] and the probiotic strains of non-pathogenic *E*. *coli* inhibiting the pathogenic *E*. *coli* K88 via production of colicin [32].

## Conclusions

The post-weaning growth check and enteric diseases including PWD are of paramount importance in the post-weaning period and continue to represent a major source of economic loss in some parts of the world’s swine industry. Allied with continuing concerns over the link between antibiotic-resistant strains of bacteria in man and the use of sub-therapeutic usage of antibiotics in livestock diets, as well as heavy metal environmental pollution, a considerable body of research and investigation into the specialized use of feed ingredients and (or) feed additives has occurred to reduce the industry’s reliance on current antimicrobial compounds. Fundamental to this research must be inquiry into the GIT of the young pig around weaning. To minimize production and economic consequences associated with the removal of in-feed antibiotics from swine diets, a search for effective alternatives/replacements to antibiotics is imperative. A number of nutritional strategies have been suggested as alternative means of enhancing post-weaning growth performance and controlling PWD in piglets.

## Competing interests

The author declares that he has no competing interests.
